# Enhanced Model for the Analysis of Thermoelectric Effects at Nanoscale: Onsager’s Method and Liu’s Technique in Comparison

**DOI:** 10.3390/e26100852

**Published:** 2024-10-09

**Authors:** Maria Di Domenico, Antonio Sellitto

**Affiliations:** Department of Industrial Engineering, University of Salerno, 84084 Fisciano, Italy; asellitto@unisa.it

**Keywords:** Liu’s technique, Onsager’s method, second law of thermodynamics, thermoelectric effects, heat waves

## Abstract

The aim of this paper is twofold. From the practical point of view, an enhanced model for the description of thermoelectric effects at nanoscale is proposed. From the theoretical point of view, instead, in the particular case of the proposed model, the equivalence between two classical techniques for the exploitation of the second law of thermodynamics is shown, i.e., Onsager’s method and Liu’s technique. An analysis of the heat-wave propagation is performed as well.

## 1. Introduction

Physics is the science which targets the study of all natural phenomena, namely, all the events that can be correctly described by means of suitable quantities displaying only clear meanings in such a way that they should be always quantifiable and/or measurable, in principle. The final goal of physics (which is not only merely academic), in particular, is the achievement of the laws (if they truly exist) which may be used to predict the evolutions (in time and in space) of the aforementioned occurrences.

Physical phenomena, indeed, may be also coupled in some circumstances: this is the case, for example, for thermoelectricity, wherein the thermal and electric phenomena are strictly related in such a way that heat can be converted into electricity, and vice versa. In this case, as well as in many others, one necessarily has to therefore couple different laws in order to obtain the so-called theoretical model, i.e., the field equations which should be employed to carry out the aimed predictions.

Generally speaking, in a theoretical model, the field equations may be made by the following three sets of equations:

1. Balance laws, i.e., the statements of the causes of the change that an extensive basic quantity (such as mass, energy, momentum, entropy and so on) undergoes over a certain domain;
2. Evolution equations, i.e., the statements of the causes of the change of intensive quantities (e.g., internal variables), and/or of *extended* physical quantities (the densities of the fluxes of the momentum and the energy, for example);
3. Constitutive equations which ensure the closure of the problem from the mathematical point of view.

The balance laws have their own clear physical roots. The same statement, however, does not always hold either for the evolution equations, or for the constitutive equations. To this end, we note that the recent decades have particularly been the witnesses of the interest in going beyond some classical formulations and/or theories of Continuum Mechanics; for example, Fourier’s law for heat conduction, Fick’s law for diffusion, and Newton’s law for viscous flow. Among the several reasons for seeking the generalizations of those laws, it is surely important to cite the well-known theoretical consequence that the introduction of the aforementioned relations into the balance equations leads to parabolic partial–differential equations, which therefore imply that the perturbations propagate with infinite speeds. This behavior is obviously at odds with the experimental evidence, and it is also disturbing from a theoretical point of view, because collective molecular effects should be expected to propagate at finite velocity, not only in a relativistic framework, but even from a non-relativistic point of view. It is currently understood that this unpleasant property can be avoided by taking into account the finite non-vanishing relaxation time of the respective fluxes (e.g., the heat flux, the diffusion flux, the momentum flux) which are sometimes also generically called dissipative fluxes. By looking at this problem from a pure mathematical point of view, one may note that a theoretical model should ultimately display a hyperbolic structure so that the actions at distance are precluded, and a finite speed of the propagation of disturbances is guaranteed.

There is also another important reason for the surge of modern theories. One should be aware, in fact, that classical formulations of Continuum Mechanics usually refer to situations which are very close to the thermodynamic equilibrium of the system. The raise of nano-technologies and their wide employments, for example, require the correct understanding of situations which are usually *very* far from equilibrium, i.e., situations wherein some well-known classical laws (as those quoted above) break down. Nano-technologies have been a real breakthrough; in the case of thermoelectric effects, for example, several research groups have recently confirmed that both one-dimensional (1D) and two-dimensional (2D) materials significantly exhibit better performances than those of the usual three-dimensional (3D) materials [1,2,3,4]. In this regard, in the literature, several interesting theoretical models for the correct description of thermoelectric effects at nano-scale appeared in recent years [5,6,7,8,9,10,11,12,13]. Since there could naturally be some discussions and concerns about the theoretical models which involve evolution and/or constitutive equations beyond the classical ones, asking what the right benchmark for them would be is a very good question. Like all the good questions, unfortunately, it does not have a unique answer.

In developing a new theoretical model, the exploitation of the second law should be (one of) the first steps to take: if the model agrees with it, in fact, then one is sure that all the mathematical solutions of the field equations (i.e., the aimed predictions) are physically acceptable [14], in principle.

In the literature, there are several techniques for testing the agreement with the second law [15,16,17,18,19,20]. Roughly speaking, the main differences among the different techniques for the exploitation of the second law lie in the starting point and the consequent final goal. In the case of Onsager’s method [15,16], for example, one initially assumes suitable constitutive relations both for the specific entropy, and for its flux: the final goal of this method, in fact, is the derivation of the (a priori unknown)evolution equations. In the case of the Coleman–Noll procedure [17] and Liu’s technique [18], as well as in their gradient extensions [19,20], however, the starting point is usually the form of the evolution equations, and the final goals are the constitutive relations for the fluxes and/or the specific entropy.

Whether those techniques are equivalent, or not, is another interesting theoretical question. We incidentally note that in Ref. [21] the authors proved that the procedures proposed by Coleman and Noll (see Ref. [17]) and by Liu (see Ref. [18]), in the case of a rigid body undergoing a temperature gradient, are equivalent in essence: they, in fact, bring about the same restrictions on the constitutive relations.

In the present paper, by assuming that the state space is

(1) $$Z\equiv \left\{z;u;q;\overset{o}{Q};Q;i\right\}$$

(refer to the Appendix A for the physical meanings of the different quantities involved), from the practical point of view, we aim to show the agreement between the following theoretical model for the analysis of thermoelectric effects at nanoscale in a rigid and isotropic body Ω

(2a) $$\begin{array}{c} \begin{array}{ccccc} \partial_{t}z=-\nabla \cdot i & \; & & \; & \forall \;X\in \Omega ,\;\forall \;t\geq 0 \end{array} \end{array}$$

(2b) $$\begin{array}{c} \begin{array}{ccccc} \partial_{t}u=-\nabla \cdot q+i\cdot E & \; & & \; & \forall \;X\in \Omega ,\;\forall \;t\geq 0 \end{array} \end{array}$$

(2c) $$\begin{array}{c} \begin{array}{ccccc} \partial_{t}q=-\frac{q}{\tau }-\left(\frac{\lambda I}{\tau }-\frac{2Q}{\theta }\right)\cdot \nabla \theta -\nabla \cdot \overset{o}{Q}-\nabla Q+\frac{\Pi }{\tau }i & \; & & \; & \forall \;X\in \Omega ,\;\forall \;t\geq 0 \end{array} \end{array}$$

(2d) $$\begin{array}{c} \begin{array}{ccccc} \partial_{t}\overset{o}{Q}\;=-\frac{\overset{o}{Q}}{\tau_{1}}-\left(\frac{2\ell^{2}}{\tau \tau_{1}}\right)\overset{o}{\nabla q^{\mathrm{sym}}} & \; & & \; & \forall \;X\in \Omega ,\;\forall \;t\geq 0 \end{array} \end{array}$$

(2e) $$\begin{array}{c} \begin{array}{ccccc} \partial_{t}Q=-\frac{Q}{\tau_{0}}-\left(\frac{5\ell^{2}}{3\tau_{0}\tau }\right)\nabla \cdot q & \; & & \; & \forall \;X\in \Omega ,\;\forall \;t\geq 0 \end{array} \end{array}$$

(2f) $$\begin{array}{c} \begin{array}{ccccc} \partial_{t}i=-\frac{i}{\tau_{e}}+\frac{\sigma_{e}}{\tau_{e}}\left(E-\epsilon \nabla \theta \right)-\frac{\epsilon \sigma_{e}}{\lambda \tau_{e}}q & \; & & \; & \forall \;X\in \Omega ,\;\forall \;t\geq 0 \end{array} \end{array}$$

and the second law of thermodynamics. Since that analysis will be performed both by Onsager’s method, and by Liu’s procedure, the above proof of compatibility will be also used here to claim that those two different techniques are equivalent in the special case of Equations (2). For the sake of clarity, in Appendix A, the physical meanings of the different quantities used throughout this paper are listed.

For the needs of this paper, we note that the form of the state space in Equation (1) is compatible with the Extended Irreversible Thermodynamics approach [22,23] which is based upon the assumption that the entropy may not only depend on the classical variables, but also on the fluxes [23,24,25,26]. As a consequence of Equation (1), in particular, in this paper, the Gibbs relation (which turns out the differential form of the state function *s*) will be generalized as

(3) $$\begin{array}{cc} ds=\left(\frac{\partial s}{\partial z}\right)dz+\left(\frac{\partial s}{\partial u}\right)du+\left(\frac{\partial s}{\partial q}\right)\cdot dq+\left(\frac{\partial s}{\partial \overset{o}{Q}}\right)\cdot d\overset{o}{Q}+\left(\frac{\partial s}{\partial Q}\right)dQ+\left(\frac{\partial s}{\partial i}\right)\cdot di\\ \; & \;\forall \;X\in \Omega ,\;\forall \;t\geq 0 \end{array}$$

For the sake of clarity, we note that Equations (2a) and (2b) are (the consequences of) balance laws, whereas Equations (2c)–(2f) have to be evolution equations. We also note that the net current into every volume V⊂Ω is given by $-\int_{\partial V}i\cdot \;dS$, with dS as the outward pointing normal of the boundary of *V*. Furthermore, the net current is also defined as the derivative in time of the total charge, which can be described as $\int_{V}\;z\;dV$. Applying the divergence theorem, Equation (2a) is obtained. In particular, Equations (2c) and (2f) can be seen as possible generalizations (see, for example, Ref. [27]) of the first thermoelectric model proposed by William Thomson (Lord Kelvin) in the XIX century [28].

The layout of the paper is as follows. In Section 2, the compatibility between Equations (2) and the second law is analyzed by means of Onsager’s method [15,16]. In Section 3, the compatibility between Equations (2) and the second law is analyzed by means of Liu’s procedure [18]. In Section 4, it is shown that Equations (2) constitute a hyperbolic system. In Section 5, both final remarks are given, and the physical quantities involved in this paper are summarized in Table A1.

## 2. Onsager’s Method

In this section, we test the agreement of the theoretical model in Equations (2) with the second law of thermodynamics by means of Onsager’s method [15,16]. As previously observed, the final goal of that method is the derivation of the evolution equations, i.e., the field equations which allow us to predict the evolutions of the extended field variables of a theoretical model: for this reason, therefore, in this section, we will properly derive Equations (2c)–(2f) in agreement with the second law, whereas Equations (2a) and (2b) simply arise from the local balance of the charge, and of the energy, respectively.

The first steps to take in Onsager’s method for the exploitation of the second law are the assumptions both about the forms of the different coefficients of Equation (3), and about the constitutive equation for the specific-entropy flux; therefore, here, we start by assuming the following constitutive relations

(4a) $$\begin{array}{cc} \; & \begin{array}{cccccccccc} \frac{\partial s}{\partial z}=\epsilon & \; & & \; & & \; & & \; & & \;\;\forall \;X\in \Omega ,\;\forall \;t\geq 0 \end{array} \end{array}$$

(4b) $$\begin{array}{cc} \; & \begin{array}{cccccccccc} \frac{\partial s}{\partial u}=\frac{1}{\theta } & \; & & \; & & \; & & \; & & \forall \;X\in \Omega ,\;\forall \;t\geq 0 \end{array} \end{array}$$

(4c) $$\begin{array}{cc} \; & \begin{array}{cccccc} \frac{\partial s}{\partial q}=-\left(\frac{\tau }{\lambda \theta^{2}}\right)q & \; & & \; & & \;\forall \;X\in \Omega ,\;\forall \;t\geq 0 \end{array} \end{array}$$

(4d) $$\begin{array}{cc} \; & \begin{array}{cccc} \frac{\partial s}{\partial \overset{o}{Q}}=-\left(\frac{\tau_{1}\tau^{2}}{2\lambda \ell^{2}\theta^{2}}\right)\overset{o}{Q} & \; & & \;\forall \;X\in \Omega ,\;\forall \;t\geq 0 \end{array} \end{array}$$

(4e) $$\begin{array}{cc} \; & \begin{array}{cccc} \frac{\partial s}{\partial Q}=-\left(\frac{3\tau_{0}\tau^{2}}{5\lambda \ell^{2}\theta^{2}}\right)Q & \; & & \;\forall \;X\in \Omega ,\;\forall \;t\geq 0 \end{array} \end{array}$$

(4f) $$\begin{array}{cc} \; & \begin{array}{ccccccc} \frac{\partial s}{\partial i}=-\left(\frac{\tau_{e}}{\sigma_{e}\theta }\right)i & \; & & \; & & \; & \forall \;X\in \Omega ,\;\forall \;t\geq 0 \end{array} \end{array}$$

which involve the form of the specific entropy, and

(5) $$\begin{array}{cccc} J^{\left(s\right)}=\frac{q}{\theta }-\frac{\tau }{\lambda \theta^{2}}\left(\overset{o}{Q}+QI\right)\cdot q+\frac{\Pi }{\theta }i & \; & & \forall \;X\in \Omega ,\;\forall \;t\geq 0 \end{array}$$

which instead gives the form of the specific-entropy flux. Recalling that the local balance of the specific entropy reads

(6) $$\begin{array}{cccccc} \partial_{t}s+\nabla \cdot J^{\left(s\right)}=\sigma^{\left(s\right)} & \; & & \; & & \forall \;X\in \Omega ,\;\forall \;t\geq 0 \end{array}$$

then the use of the other two balance laws of our model, i.e., Equations (2a) and (2b), allows us to straightforwardly obtain

(7) $$\begin{array}{cc} \sigma^{\left(s\right)}= & -\frac{\tau }{\lambda \theta^{2}}\left[\partial_{t}q+\frac{\lambda }{\tau }\nabla \theta -\frac{2}{\theta }\left(QI+\overset{o}{Q}\right)\cdot \nabla \theta +\nabla \cdot \overset{o}{Q}+\nabla Q-\frac{\Pi }{\tau }i\right]\cdot q\\ \; & -\frac{\tau^{2}\tau_{1}}{2\lambda \ell^{2}\theta^{2}}\left(\partial_{t}\overset{o}{Q}+\frac{2\ell^{2}}{\tau \tau_{1}}\overset{o}{\nabla q^{\mathrm{sym}}}\right)\cdot \overset{o}{Q}-\frac{3\tau^{2}\tau_{0}}{5\lambda \ell^{2}\theta^{2}}\left(\partial_{t}Q+\frac{5\ell^{2}}{3\tau \tau_{0}}\nabla \cdot q\right)Q\\ \; & -\frac{\tau_{e}}{\sigma_{e}\theta }\left[\partial_{t}i-\frac{\sigma_{e}}{\tau_{e}}\left(E-\frac{\Pi }{\theta }\nabla \theta \right)+\frac{\Pi \sigma_{e}}{\lambda \tau_{e}\theta }q\right]\cdot i+\left(\frac{\Pi }{\theta }-\epsilon \right)\nabla \cdot i \end{array}$$

∀X∈Ω and ∀t≥0, once the quantity $\left(\frac{\Pi }{\lambda \theta^{2}}\right)q\cdot i$ has been added, and subtracted, in the left-hand side of Equation (6) for our purposes.

From Equation (7) it can be argued that in our case the specific-entropy production is a linear combination of the thermodynamic fluxes q, $\overset{o}{Q}$, *Q* and i, and the following thermodynamic forces

(8a) $$\begin{array}{cc} \; & f^{q}:=\partial_{t}q+\frac{\lambda }{\tau }\nabla \theta -\frac{2}{\theta }\left(QI+\overset{o}{Q}\right)\cdot \nabla \theta +\nabla \cdot \overset{o}{Q}+\nabla Q-\frac{\Pi }{\tau }i \end{array}$$

(8b) $$\begin{array}{cc} \; & F^{Q}:=\partial_{t}\overset{o}{Q}+\frac{2\ell^{2}}{\tau \tau_{1}}\overset{o}{\nabla q^{\mathrm{sym}}} \end{array}$$

(8c) $$\begin{array}{cc} \; & F^{Q}:=\partial_{t}Q+\frac{5\ell^{2}}{3\tau \tau_{0}}\nabla \cdot q \end{array}$$

(8d) $$\begin{array}{cc} \; & f^{i}:=\partial_{t}i-\frac{\sigma_{e}}{\tau_{e}}\left(E-\frac{\Pi }{\theta }\nabla \theta \right)+\frac{\Pi \sigma_{e}}{\lambda \tau_{e}\theta }q \end{array}$$

which appear therein. In order that the thermodynamic unilateral constraint $\sigma^{\left(s\right)}$ is always (i.e., ∀X∈Ω and ∀t≥0) fulfilled, it is sufficient to assume

(9a) $$\begin{array}{cc} \; & \begin{array}{ccccccc} \frac{q}{\tau }=-f^{q} & \; & & \; & & \; & \forall \;X\in \Omega ,\;\forall \;t\geq 0 \end{array} \end{array}$$

(9b) $$\begin{array}{cc} \; & \begin{array}{cccccc} \frac{\overset{o}{Q}}{\tau_{1}}=-F^{Q} & \; & & \; & & \forall \;X\in \Omega ,\;\forall \;t\geq 0 \end{array} \end{array}$$

(9c) $$\begin{array}{cc} \; & \begin{array}{cccccc} \frac{Q}{\tau_{0}}=-F^{Q} & \; & & \; & & \forall \;X\in \Omega ,\;\forall \;t\geq 0 \end{array} \end{array}$$

(9d) $$\begin{array}{cc} \; & \begin{array}{ccccccc} \frac{i}{\tau_{e}}=-f^{i} & \; & & \; & & \; & \forall \;X\in \Omega ,\;\forall \;t\geq 0 \end{array} \end{array}$$

together with the constitutive assumption

(10) $$\epsilon =\frac{\Pi }{\theta }$$

which is sometimes referred to as the *second Kelvin relation*. The final insertion of Equations (9) into Equation (8) directly yields the evolution equations we were searching for here, namely Equations (2c)–(2f).

The above thermodynamic considerations, therefore, allow us to claim that the theoretical model in Equation (2) agrees with the second law of thermodynamics.

We note that Equations (9), referring to the linear relation between the fluxes and the forces [22], represent the simplest way to ensure that $\sigma^{\left(s\right)}\geq 0$. In this case, in fact, we have

(11) $$\begin{array}{cc} \sigma^{\left(s\right)}=\left(\frac{1}{\lambda \theta^{2}}\right)q\cdot q+\left(\frac{\tau^{2}}{2\lambda \ell^{2}\theta^{2}}\right)\overset{o}{Q}\cdot \overset{o}{Q}+\left(\frac{3\tau^{2}}{5\lambda \ell^{2}\theta^{2}}\right)Q^{2}+\left(\frac{1}{\sigma_{e}\theta }\right)i\cdot i\\ \; & \;\forall \;X\in \Omega ,\;\forall \;t\geq 0 \end{array}$$

Other sufficient (and more refined) conditions could be obtained by allowing, for example, the thermodynamic forces to non-linearly depend on the fluxes [23].

In closing this section, we note that Equations (4) is tantamount to assume the following constitutive equation for *s*

(12) $$\begin{array}{cc} s=s_{0}\left(u\right)+\epsilon z-\left(\frac{\tau }{2\lambda \theta^{2}}\right)q\cdot q-\left(\frac{\tau_{1}\tau^{2}}{4\lambda \ell^{2}\theta^{2}}\right)\;\overset{o}{Q}\cdot \overset{o}{Q}-\left(\frac{3\tau_{0}\tau^{2}}{10\lambda \ell^{2}\theta^{2}}\right)Q^{2}-\left(\frac{\tau_{e}}{2\sigma_{e}\theta }\right)i\cdot i\\ \; & \;\forall \;X\in \Omega ,\;\forall \;t\geq 0 \end{array}$$

wherein $s_{0}\left(u\right)$ stands for the part of the local-equilibrium specific entropy which only depends on the internal energy (or the temperature).

## 3. Liu’s Procedure

In this section, we test the agreement of the theoretical model in Equations (2) with the second law of thermodynamics by means of Liu’s procedure [18].

From a practical point of view, we initially admit here the validity of the model Equations (2), and look at the constitutive relations for *s* and $J^{\left(s\right)}$, which guarantee that $\sigma^{\left(s\right)}\geq 0$, ∀X∈Ω and ∀t≥0. Liu’s technique [18], in fact, is based on the method of the Lagrange multipliers and allows us to derive necessary and sufficient conditions (that is, the aforementioned constitutive relations) ensuring that the linear combination between the specific entropy production and the field equations is always compliant with the second law of thermodynamics [29]. The coefficients of that linear combination are exactly the Lagrange multipliers. Recalling that the specific entropy production is given by the left-hand side of Equation (6), the above aimed constitutive relations have to be derived in such a way that the following constraint for the *extended* entropy inequality

(13) $$\begin{array}{cc} \; & \partial_{t}s+\nabla \cdot J^{\left(s\right)}-\Lambda^{z}\left(\partial_{t}z+\nabla \cdot i\right)-\Lambda^{u}\left(\partial_{t}u+\nabla \cdot q-i\cdot E\right)\\ \; & \;-\Lambda^{q}\cdot \left[\partial_{t}q+\frac{q}{\tau }+\left(\frac{\lambda I}{\tau }-\frac{2Q}{\theta }\right)\cdot \nabla \theta +\nabla \cdot \overset{o}{Q}+\nabla Q-\frac{\Pi }{\tau }i\right]\\ \; & \;-\Lambda^{Q}\cdot \left[\partial_{t}\overset{o}{Q}\;+\frac{\overset{o}{Q}}{\tau_{1}}+\left(\frac{2\ell^{2}}{\tau \tau_{1}}\right)\overset{o}{\nabla q^{\mathrm{sym}}}\right]-\Lambda^{Q}\left[\partial_{t}Q+\frac{Q}{\tau_{0}}+\left(\frac{5\ell^{2}}{3\tau_{0}\tau }\right)\nabla \cdot q\right]\\ \; & \;-\Lambda^{i}\cdot \left[\partial_{t}i+\frac{i}{\tau_{e}}-\frac{\sigma_{e}}{\tau_{e}}\left(E-\epsilon \nabla \theta \right)+\frac{\epsilon \sigma_{e}}{\lambda \tau_{e}}q\right]\geq 0 \end{array}$$

is fulfilled ∀X∈Ω and ∀t≥0. For the sake of clarity, we note that in the above inequality, the parameters $\Lambda^{z}$, $\Lambda^{u}$, $\Lambda^{q}$, $\Lambda^{Q}$, $\Lambda^{Q}$ and $\Lambda^{i}$ are the so-called Lagrange multipliers which, in principle, may depend on the whole set of the state variables (1). The same conclusion also holds for the constitutive equations of *s* and $J^{\left(s\right)}$.

Taking into account the usual relation $du=c_{v}d\theta$, by writing the explicit forms both of $\partial_{t}s$, and of $\nabla \cdot J^{\left(s\right)}$ in terms of the state variables, from inequality (13), one can reach the following two sets of necessary and sufficient conditions, which guarantee that second law of thermodynamics is always fulfilled

(14a) $$\begin{array}{cc} \; & \begin{array}{cccccc} \frac{\partial s}{\partial z}=\Lambda^{z} & \; & & \; & & \;\;\forall \;X\in \Omega ,\;\forall \;t\geq 0 \end{array} \end{array}$$

(14b) $$\begin{array}{cc} \; & \begin{array}{cccccc} \frac{\partial s}{\partial u}=\Lambda^{u} & \; & & \; & & \;\forall \;X\in \Omega ,\;\forall \;t\geq 0 \end{array} \end{array}$$

(14c) $$\begin{array}{cc} \; & \begin{array}{cccccc} \frac{\partial s}{\partial q}=\Lambda^{q} & \; & & \; & & \;\forall \;X\in \Omega ,\;\forall \;t\geq 0 \end{array} \end{array}$$

(14d) $$\begin{array}{cc} \; & \begin{array}{ccccc} \frac{\partial s}{\partial \overset{o}{Q}}=\Lambda^{Q} & \; & & \; & \;\;\;\;\forall \;X\in \Omega ,\;\forall \;t\geq 0 \end{array} \end{array}$$

(14e) $$\begin{array}{cc} \; & \begin{array}{ccccc} \frac{\partial s}{\partial Q}=\Lambda^{Q} & \; & & \; & \;\;\;\;\forall \;X\in \Omega ,\;\forall \;t\geq 0 \end{array} \end{array}$$

(14f) $$\begin{array}{cc} \; & \begin{array}{cccccc} \frac{\partial s}{\partial i}=\Lambda^{i} & \; & & \; & & \;\;\;\forall \;X\in \Omega ,\;\forall \;t\geq 0 \end{array} \end{array}$$

and

(15a) $$\begin{array}{cc} \; & \begin{array}{ccccccccccccccc} \frac{\partial J^{\left(s\right)}}{\partial z}=0 & \; & & \; & & \; & & \; & & \; & & \; & & \; & \;\;\forall \;X\in \Omega ,\;\forall \;t\geq 0 \end{array} \end{array}$$

(15b) $$\begin{array}{cc} \; & \begin{array}{ccccc} \frac{\partial J^{\left(s\right)}}{\partial \theta }=\left[\frac{\lambda }{\tau }I-\frac{2}{\theta }\left(\overset{o}{Q}+QI\right)\right]\cdot \Lambda^{q}+\frac{\sigma_{e}\epsilon }{\tau_{e}}\Lambda^{i} & \; & & \; & \forall \;X\in \Omega ,\;\forall \;t\geq 0 \end{array} \end{array}$$

(15c) $$\begin{array}{cc} \; & \begin{array}{ccccc} \frac{\partial J^{\left(s\right)}}{\partial q}=\Lambda^{u}I+\left(\frac{2\ell^{2}}{\tau \tau_{1}}\right)\Lambda^{Q}+\left(\frac{5\ell^{2}}{3\tau \tau_{0}}\right)\Lambda^{Q}I & \; & & \; & \;\;\;\forall \;X\in \Omega ,\;\forall \;t\geq 0 \end{array} \end{array}$$

(15d) $$\begin{array}{cc} \; & \begin{array}{cccccccccccccc} \frac{\partial J^{\left(s\right)}}{\partial \overset{o}{Q}}=\Lambda^{q}I & \; & & \; & & \; & & \; & & \; & & \; & & \;\;\forall \;X\in \Omega ,\;\forall \;t\geq 0 \end{array} \end{array}$$

(15e) $$\begin{array}{cc} \; & \begin{array}{ccccccccccccccc} \frac{\partial J^{\left(s\right)}}{\partial Q}=\Lambda^{q} & \; & & \; & & \; & & \; & & \; & & \; & & \; & \forall \;X\in \Omega ,\;\forall \;t\geq 0 \end{array} \end{array}$$

(15f) $$\begin{array}{cc} \; & \begin{array}{ccccccccccccccc} \frac{\partial J^{\left(s\right)}}{\partial i}=\Lambda^{z}I & \; & & \; & & \; & & \; & & \; & & \; & & \; & \forall \;X\in \Omega ,\;\forall \;t\geq 0 \end{array} \end{array}$$

together with the *reduced* entropy inequality

(16) $$\begin{array}{c} \sigma^{\left(s\right)}=\Lambda^{u}E\cdot i-\Lambda^{q}\cdot \left(\frac{q}{\tau }-\frac{\Pi }{\tau }i\right)-\Lambda^{Q}\cdot \frac{\overset{o}{Q}}{\tau_{1}}-\Lambda^{Q}\frac{Q}{\tau_{0}}-\Lambda^{i}\cdot \left(\frac{i}{\tau_{e}}-\frac{\sigma_{e}}{\tau_{e}}E+\frac{\sigma_{e}\epsilon }{\lambda \tau_{e}}q\right)\geq 0\\ \forall \;X\in \Omega ,\;\forall \;t\geq 0 \end{array}$$

If the following identifications are made

(17a) $$\begin{array}{cc} \; & \Lambda^{z}:=\frac{\Pi }{\theta } \end{array}$$

(17b) $$\begin{array}{cc} \; & \Lambda^{u}:=\frac{1}{\theta } \end{array}$$

(17c) $$\begin{array}{cc} \; & \Lambda^{q}:=-\left(\frac{\tau }{\lambda \theta^{2}}\right)q \end{array}$$

(17d) $$\begin{array}{cc} \; & \Lambda^{Q}:=-\left(\frac{\tau_{1}\tau^{2}}{2\lambda \ell^{2}\theta^{2}}\right)\;\overset{o}{Q} \end{array}$$

(17e) $$\begin{array}{cc} \; & \Lambda^{Q}:=-\left(\frac{3\tau_{0}\tau^{2}}{5\lambda \ell^{2}\theta^{2}}\right)Q \end{array}$$

(17f) $$\begin{array}{cc} \; & \Lambda^{i}:=-\left(\frac{\tau_{e}}{\sigma_{e}\theta }\right)i \end{array}$$

then, based on the previous thermodynamic restrictions, it can be easily inferred that the theoretical model described by Equations (2) agrees with the second law if

- the specific entropy is given by Equation (12);
- the specific-entropy flux is given by Equation (5).

Equations (12) and (5) arise, respectively, from the integration of Equations (14) and (15) (details can be found in Appendix B).

By coupling Equations (16) and (17), it is also straightforward to obtain that the specific-entropy production is still given by Equation (11).

## 4. The Propagation Speed of Waves

We devote this section to pointing out the hyperbolic character of the theoretical model in Equations (2).

In order to find the speed of the propagation of the waves predicted by the equation, here, we employ the tool of the acceleration waves in particular; that is, we assume the presence in our system of a traveling surface S across which the state variables are continuous, whereas all their derivatives (both in time, and in space) display at most finite discontinuities. From the mathematical point of view, an acceleration wave may model, for example, the thermal perturbation generated by a periodic variation in time of the temperature in a point of the system with respect to its steady-state reference level.

For the sake of simplicity, here, we also suppose that S is moving into equilibrium zones, that is, we assume that the region $\Omega^{★}$ ahead of S is such that

(18) $$\begin{array}{cccccc} z=z_{0}\;\theta =\theta_{0}\;q=q_{0}\;Q=Q_{0}\;i=i_{0} & \; & & \; & & \forall \;X\in \Omega^{★},\;\forall \;t\geq 0 \end{array}$$

with $z_{0}$, $\theta_{0}$, $q_{0}$, $Q_{0}$ and $i_{0}$ being the stationary reference levels. If all the material functions are constant (i.e., if they only display vanishingly small variations when the temperature of the system changes), then from Equations (2) we may directly obtain

(19a) $$\begin{array}{cc} \; & ⟦\partial_{t}z⟧=-⟦\nabla \cdot i⟧ \end{array}$$

(19b) $$\begin{array}{cc} \; & ⟦\partial_{t}u⟧=-⟦\nabla \cdot q⟧ \end{array}$$

(19c) $$\begin{array}{cc} \; & ⟦\partial_{t}q⟧=-\left(\frac{\lambda I}{\tau }-\frac{2Q_{0}}{\theta_{0}}\right)⟦\nabla \theta ⟧-⟦\nabla \cdot \overset{o}{Q}⟧-⟦\nabla Q⟧ \end{array}$$

(19d) $$\begin{array}{cc} \; & ⟦\partial_{t}\overset{o}{Q}\;⟧=-\left(\frac{2\ell^{2}}{\tau \tau_{1}}\right)⟦\overset{o}{\nabla q^{\mathrm{sym}}}⟧ \end{array}$$

(19e) $$\begin{array}{cc} \; & ⟦\partial_{t}Q⟧=-\left(\frac{5\ell^{2}}{3\tau_{0}\tau }\right)⟦\nabla \cdot q⟧ \end{array}$$

(19f) $$\begin{array}{cc} \; & ⟦\partial_{t}i⟧=-\frac{\sigma_{e}\epsilon }{\tau_{e}}⟦\nabla \theta ⟧ \end{array}$$

Making use of the classical Hadamard relations [30,31]

(20a) $$\begin{array}{cc} \; & ⟦\partial_{t}z⟧=-U_{N}\hat{z} \end{array}$$

(20b) $$\begin{array}{cc} \; & ⟦\partial_{t}\theta ⟧=-U_{N}\hat{\theta } \end{array}$$

(20c) $$\begin{array}{cc} \; & ⟦\partial_{t}q⟧=-U_{N}\hat{q} \end{array}$$

(20d) $$\begin{array}{cc} \; & ⟦\partial_{t}\overset{o}{Q}⟧=-U_{N}\hat{\overset{o}{Q}} \end{array}$$

(20e) $$\begin{array}{cc} \; & ⟦\partial_{t}Q⟧=-U_{N}\hat{Q} \end{array}$$

(20f) $$\begin{array}{cc} \; & ⟦\partial_{t}i⟧=-U_{N}\hat{i} \end{array}$$

wherein $\hat{z}=n\cdot ⟦\nabla z⟧$, $\hat{\theta }=n\cdot ⟦\nabla \theta ⟧$, $\hat{q}=n\cdot ⟦\nabla q⟧$, $\hat{\overset{o}{Q}}=n\cdot ⟦\nabla \overset{o}{Q}⟧$, $\hat{Q}=n\cdot ⟦\nabla Q⟧$ and $\hat{i}=n\cdot ⟦\nabla i⟧$ are the wave amplitudes, with n being the normal unit to S, and $U_{N}$ the non-vanishing wave speed, by direct computations from Equations (19), the following system of homogeneous algebraic equations holds

(21a) $$\begin{array}{cc} \; & U_{N}\hat{z}-n\cdot \hat{i}=0 \end{array}$$

(21b) $$\begin{array}{cc} \; & U_{N}\hat{\theta }-\frac{1}{c_{v}}n\cdot \hat{q}=0 \end{array}$$

(21c) $$\begin{array}{cc} \; & U_{N}\hat{q}-\left(\frac{\lambda }{\tau }n-\frac{2}{\theta_{0}}Q_{0}\cdot n\right)\hat{\theta }-\hat{\overset{o}{Q}}\cdot n-\hat{Q}n=0 \end{array}$$

(21d) $$\begin{array}{cc} \; & U_{N}\hat{\overset{o}{Q}}-\frac{4\ell^{2}}{3\tau_{1}\tau }\hat{q}\;n=0 \end{array}$$

(21e) $$\begin{array}{cc} \; & U_{N}\hat{Q}-\frac{5\ell^{2}}{3\tau_{0}\tau }\hat{q}\cdot n=0 \end{array}$$

(21f) $$\begin{array}{cc} \; & U_{N}\hat{i}-\frac{\sigma_{e}\epsilon }{\tau_{e}}\hat{\theta }\;n=0 \end{array}$$

In order to avoid Sys. (21) only admitting the trivial solution for the wave amplitudes, the following relation has to hold

(22) $$U_{N}^{4}\left[U_{N}^{2}-U_{\mathrm{mc}}^{2}\left(1+\chi_{1}+\chi_{2}-\chi_{3}\right)\right]=0$$

wherein

(23) $$U_{\mathrm{mc}}=\sqrt{\frac{\lambda }{\tau c_{v}}}$$

is the second-sound speed in the Maxwell–Cattaneo theory, and

(24a) $$\begin{array}{cc} \; & \chi_{1}=\frac{4\ell^{2}c_{v}}{3\tau_{1}\lambda } \end{array}$$

(24b) $$\begin{array}{cc} \; & \chi_{2}=\frac{5\ell^{2}c_{v}}{3\tau_{0}\lambda } \end{array}$$

(24c) $$\begin{array}{cc} \; & \chi_{3}=\frac{2\tau \;\mathrm{tr}\left(Q_{0}\right)}{\lambda \theta_{0}} \end{array}$$

are three dimensionless parameters accounting for the non-local effects (through $\ell^{2}$), the relaxational effects (through the relaxation times of the second-order fluxes), and the stationary reference values for the temperature and the second-order flux, respectively. By avoiding the trivial solution $U_{N}=0$, Equation (22) allows us to claim that the speed of propagation predicted by the theoretical model in Equations (2) is given by

(25) $$U_{N}=U_{\mathrm{mc}}\sqrt{1+\chi_{1}+\chi_{2}-\chi_{3}}$$

According to Equation (25), below we list some brief comments about the role played by non-local and non-linear effects.

- The role of the non-local effects is introduced by $\chi_{1}$ and $\chi_{2}$; since those parameters are always positive, it is possible to claim that non-local effects always enhance the speed of propagation with respect to $U_{\mathrm{mc}}$.
- The role of the non-linear effects is introduced by $\chi_{3}$; since it may attain, in principle, both positive and negative values, we can claim that non-linear effects can either enhance or reduce the speed of propagation with respect to $U_{\mathrm{mc}}$.
- Whenever both the non-local effects, and the non-linear ones, are negligible in Equations (2), then the predicted speed of propagation (25) reduces to $U_{\mathrm{mc}}$.

## 5. Final Remarks

The possibility of converting the heat into the electric current, and vice versa, is one of the best-known coupled transport phenomena since its discovery two centuries ago. Notwithstanding this, in the past, thermoelectric devices practically achieved a scant success; this was largely the consequence of their very low efficiencies. The rise of nano-technologies has been a real breakthrough for thermoelectricity, because they allow us to currently develop new thermoelectric materials [32,33,34]; by obtaining the device’s sizes comparable to the mean-free path of the different heat carriers (phonons, electrons, holes, etc.), in fact, it is now possible to significantly enhance the thermoelectric features of a material.

The research regarding thermoelectricity, however, should not only be limited to the search of new materials, but it should also look at enhanced and even more refined theoretical models for the correct description of the thermoelectric phenomenon, in accordance with the essence of physics. For this reason, in the present paper, we proposed the model in Equations (2) for the description of thermoelectric effects at nano-scale. That model has the main merit of giving emphasis to non-local and non-linear effects, the influence of which has been especially pointed out here on the speed of propagation of thermal waves.

We note that the aforementioned model neither arises from a rigorous microscopic derivation, nor lies on experimental observations: it is therefore currently meant only as theoretical proposal. In order to give a physical value to Equations (2), however, in Section 2 and Section 3 we scrutinized them in view of the second law of thermodynamics. That analysis has been performed by means of two different procedures: both of them turned out the compatibility of the theoretical model described by Equations (2) with the basic tenets of Continuum Mechanics. The study of the waves’ propagation of Section 4 also indicates that the theoretical model (2) is hyperbolic, namely, it is compatible with the physical evidence that mechanical disturbances can only propagate with a finite speed.

By looking at a future analysis for the rigorous proof, we close this paper by observing that the perfect agreement between the results contained in Section 2 and those of Section 3 could be the first evidence of the possible equivalence between Onsager’s method [15,16] and Liu technique [18].

## Data Availability

Data are contained within the article.

## Appendix A

**Table A1:** List of the physical quantities.

Symbol	Meaning	Unit of Measurement
θ	non-equilibrium temperature	K
$\theta_{0}$	reference value of temperature	K
*u*	specific internal energy	$\frac{J}{m^{3}}$
q	local heat flux	$\frac{W}{m^{2}}$
$q_{0}$	reference value of the local heat flux	$\frac{W}{m^{2}}$
Q	flux of the local heat flux	$\frac{W}{m\;s}$
$Q_{0}$	reference value of the flux of the local heat flux	$\frac{W}{m\;s}$
$\overset{o}{Q}$	deviatoric (traceless) part of Q	$\frac{W}{m\;s}$
*Q*	volumetric part of Q	$\frac{W}{m\;s}$
*z*	charge per unit volume of electrons	$\frac{C}{m^{3}}$
$z_{0}$	reference value of the charge per unit volume of electrons	$\frac{C}{m^{3}}$
i	electrical-current density	$\frac{C}{m^{2}s}$
$i_{0}$	reference value of the electrical-current density	$\frac{C}{m^{2}s}$
E	electric field	$\frac{N}{C}$
τ	relaxation time of the heat carriers	s
$\tau_{e}$	relaxation time of electrons	s
$\tau_{1}$	relaxation time of $\overset{o}{Q}$	s
$\tau_{0}$	relaxation time of *Q*	s
*t*	time	s
λ	thermal conductivity	$\frac{W}{m\;K}$
*ℓ*	mean-free path of the heat carriers	m
X	position vector	m
$c_{v}$	specific heat at constant volume	$\frac{J}{m^{3}K}$
ϵ	Seebeck coefficient	$\frac{J}{K\;C}$
Π	Peltier coefficient	$\frac{J}{C}$
$\sigma_{e}$	electrical conductivity	$\frac{C^{2}}{J\;m\;s}$
*s*	specific entropy	$\frac{J}{m^{3}K}$
$J^{s}$	specific-entropy flux	$\frac{W}{m^{2}K}$
$\sigma^{s}$	specific-entropy production	$\frac{W}{m^{3}K}$
$\Lambda^{z}$	Lagrange multiplier	$\frac{J}{K\;C}$
$\Lambda^{u}$	Lagrange multiplier	$\frac{1}{K}$
$\Lambda^{q}$	Lagrange multiplier	$\frac{s}{m\;K}$
$\Lambda^{Q}$	Lagrange multiplier	$\frac{s}{m^{2}K}$
$\Lambda^{Q}$	Lagrange multiplier	$\frac{s}{m^{2}K}$
$\Lambda^{i}$	Lagrange multiplier	$\frac{C}{m^{2}\;s\;K}$
$U_{N}$	propagation speed	$\frac{m}{s}$
$U_{\mathrm{mc}}$	propagation speed in the Maxwell–Cattaneo theory	$\frac{m}{s}$
$f^{q}$	Thermodynamic force	$\frac{W}{m^{2}s}$
$F^{q}$	Thermodynamic force	$\frac{W}{ms^{2}}$
$F^{Q}$	Thermodynamic force	$\frac{W}{ms^{2}}$
$f^{i}$	Thermodynamic force	$\frac{C}{m^{2}s^{2}}$

## Appendix B. Integration of Equations (15)

The aim of this section is to give details about how Equation (5) arises from the integration of Equations (15), together with the constitutive assumption (10).

Starting from Equation (15a), we infer that $J^{s}=J^{s}\left(\theta ,q,\overset{o}{Q},Q,i\right)$. Assuming Equations (17), Equations (15) become

(A1a) $$\begin{array}{cc} \; & \begin{array}{ccccccccccccccc} \frac{\partial J^{\left(s\right)}}{\partial z}=0 & \; & & \; & & \; & & \; & & \; & & \; & & \; & \;\;\forall \;X\in \Omega ,\;\forall \;t\geq 0 \end{array} \end{array}$$

(A1b) $$\begin{array}{cc} \; & \begin{array}{ccc} \frac{\partial J^{\left(s\right)}}{\partial \theta }=\left(-\frac{\tau }{\lambda \theta^{2}}\right)\left[\frac{\lambda }{\tau }I-\frac{2}{\theta }\left(\overset{o}{Q}+QI\right)\right]\cdot q-\frac{\epsilon }{\theta }i & \; & \;\;\forall \;X\in \Omega ,\;\forall \;t\geq 0 \end{array} \end{array}$$

(A1c) $$\begin{array}{cc} \; & \begin{array}{ccccccc} \frac{\partial J^{\left(s\right)}}{\partial q}=\frac{1}{\theta }I-\left(\frac{\tau }{\lambda \theta^{2}}\right)\overset{o}{Q}-\left(\frac{\tau }{\lambda \theta^{2}}\right)QI & \; & & \; & & \; & \;\;\;\;\;\forall \;X\in \Omega ,\;\forall \;t\geq 0 \end{array} \end{array}$$

(A1d) $$\begin{array}{cc} \; & \begin{array}{cccccccccccccc} \frac{\partial J^{\left(s\right)}}{\partial \overset{o}{Q}}=-\frac{\tau }{\lambda \theta^{2}}qI & \; & & \; & & \; & & \; & & \; & & \; & & \forall \;X\in \Omega ,\;\forall \;t\geq 0 \end{array} \end{array}$$

(A1e) $$\begin{array}{cc} \; & \begin{array}{cccccccccccccc} \frac{\partial J^{\left(s\right)}}{\partial Q}=-\frac{\tau }{\lambda \theta^{2}}q & \; & & \; & & \; & & \; & & \; & & \; & & \;\;\;\forall \;X\in \Omega ,\;\forall \;t\geq 0 \end{array} \end{array}$$

(A1f) $$\begin{array}{cc} \; & \begin{array}{cccccccccccccccc} \frac{\partial J^{\left(s\right)}}{\partial i}=\frac{\Pi }{\theta }I & \; & & \; & & \; & & \; & & \; & & \; & & \; & & \;\;\forall \;X\in \Omega ,\;\forall \;t\geq 0 \end{array} \end{array}$$

Equation (A1f) leads to

(A2) $$J^{s}=\frac{\Pi }{\theta }i+\alpha_{1}\left(\theta ,q,\overset{o}{Q},Q\right)$$

with $\alpha_{1}$ as a suitable vectorial function of the aforementioned variables. Substituting the previous equation into Equation (A1e), one can reach

(A3) $$\alpha_{1}=-\frac{\tau }{\lambda \theta^{2}}Qq+\alpha_{2}\left(\theta ,q,\overset{o}{Q}\right)$$

The coupling between Equations (A2) and (A3), together with Equation (A1d), furnishes

(A4) $$J^{s}=\frac{\Pi }{\theta }i-\frac{\tau }{\lambda \theta^{2}}\left(\overset{o}{Q}+QI\right)\cdot q+\alpha_{3}\left(\theta ,q\right)$$

Substituting Equation (A4) into Equation (A1c) one obtains

(A5) $$\alpha_{3}=\frac{q}{\theta }+\alpha_{4}\left(\theta \right)$$

Finally, taking into account Equations (A5), (A4) and (A1b), the requested Expression (5) for the entropy flux arises.
